# Theoretical Basis for Dynamic Label Propagation in Stationary Metabolic Networks under Step and Periodic Inputs

**DOI:** 10.1371/journal.pone.0144652

**Published:** 2015-12-07

**Authors:** Serguei Sokol, Jean-Charles Portais

**Affiliations:** 1 Laboratoire d’Ingénierie des Systèmes Biologiques et des Procédés, LISBP, Université de Toulouse, INSA, UPS, INP, Toulouse, France; 2 Laboratoire Ingénierie des Systèmes Biologiques et des Procédés, INRA UMR792, Toulouse, France; 3 UMR5504, CNRS, Toulouse, France; University of Erlangen-Nuremberg, GERMANY

## Abstract

The dynamics of label propagation in a stationary metabolic network during an isotope labeling experiment can provide highly valuable information on the network topology, metabolic fluxes, and on the size of metabolite pools. However, major issues, both in the experimental set-up and in the accompanying numerical methods currently limit the application of this approach. Here, we propose a method to apply novel types of label inputs, sinusoidal or more generally periodic label inputs, to address both the practical and numerical challenges of dynamic labeling experiments. By considering a simple metabolic system, i.e. a linear, non-reversible pathway of arbitrary length, we develop mathematical descriptions of label propagation for both classical and novel label inputs. Theoretical developments and computer simulations show that the application of rectangular periodic pulses has both numerical and practical advantages over other approaches. We applied the strategy to estimate fluxes in a simulated experiment performed on a complex metabolic network (the central carbon metabolism of *Escherichia coli*), to further demonstrate its value in conditions which are close to those in real experiments. This study provides a theoretical basis for the rational interpretation of label propagation curves in real experiments, and will help identify the strengths, pitfalls and limitations of such experiments. The cases described here can also be used as test cases for more general numerical methods aimed at identifying network topology, analyzing metabolic fluxes or measuring concentrations of metabolites.

## Introduction

Isotopic studies of biochemical systems have been greatly improved since they were first used to identify metabolic pathways in the early 1930s. Modern approaches combine isotope labeling experiments with mathematical models to obtain highly detailed quantitative information about metabolic pathways (particularly metabolic fluxes) and networks of increasing size and complexity [1, 2]. From a mathematical point of view, these approaches require solving two related problems: first, the ‘direct’ problem of calculating labeling distribution in metabolites when the fluxes and, where necessary, metabolite pools are known, plus the ‘inverse’ problem of calculating fluxes (and metabolite pools) from the measured label distributions. In the 2000s, in situations where the metabolic state and label distribution are stationary, both problems were solved numerically in a cumomer (*cum*ulated isotopo*mer*s) framework [3]. Since then, the numerical efficiency of solving such problems increased dramatically thanks to progresses in different aspect: reduction in the size of the direct problem which was achieved in the framework of elementary metabolite units (EMU) [4], more efficient programming [5] and better fitting algorithms such as the non-linear least squares algorithm NLSIC [6]. It now takes from only a few seconds to at the most a few minutes to solve inverse problems of flux estimation for stationary labeling versus up to several hours previously.

The current challenge is to efficiently solve both the direct and inverse problems in situations in which the metabolic context is stationary but the label distribution is not (i.e. dynamic label propagation in a stationary network). Knowing such dynamics provides significantly more information about metabolic systems [7]. An efficient mathematical solution to the direct problem, i.e. simulating dynamic label propagation is indispensable to solve the corresponding inverse problem in a reasonable time frame and with acceptable accuracy and precision. Promising results have been reported in several particular cases [8–11] and the first publicly available software was reported recently [12, 13]. However, many difficulties remain to be overcome. First, direct simulation often requires finding numerical solution for ordinary differential equations (ODE). In its general form, it may be a difficult numerical problem *per se* due to approximation errors introduced by a discretization scheme or more importantly, due to numerical stability issues, not to mention the tremendous quantity of calculation that may be required. Next, the metabolite concentrations are now part of the problem and they are tightly coupled with the fluxes. This makes the inverse problem of flux and concentration estimation even more ill-conditioned than in stationary labeling. Finally, using a linearized approach for the minimization of non-linear least squares can require even more iterations due to the increased non-linearity of the problem.

In this context, it is important to find theoretical solutions to properly describe label propagation in stationary metabolic networks. Here, we report on two major developments in the field. First, we provide analytical solutions to several basic problems in dynamic ^13^C-labeling experiments. These solutions were evaluated using case studies of increasing difficulty and generality. They provide a better understanding of the mechanisms underlying label propagation but also of the nature of the difficulties that have to be addressed in any past or future numerical approaches like the appearance of singularities, sources of non-linearity, non-identifiability etc. Second, instead of the step label input classically applied in dynamic labeling experiments, we introduce novel types of label inputs: sinusoidal and rectangular periodic pulses (RPP), as useful strategies to investigate dynamically labeled stationary systems.

The paper is organized as follows. The following Analysis section comprises five subsections. In the first subsection, the label propagation problem is formulated. Analytical solutions are then provided for linear non-reversible pathways of arbitrary length. The solutions for this pathway are analyzed for three types of label inputs: 1) the classical step label experiment (also known as label shift or jump); 2) a sinusoidal label wave and 3) an RPP labeling. In the fifth subsection, we propose an approach for studying a complex metabolic network in the context of RPP labeling. In the last section Results and Discussion, we discuss a numerical experiment on a complex network inspired by a real world example.

A script written in R (http://www.R-project.org) to enable modeling of all the cases examined here as well as all the necessary data is freely available under an OpenSource licence (cf. S1 Software).

## Analysis

### Problem formulation

An ODE describing the balance of a metabolite quantity *m* is conceptually simple and can be written as

$$\begin{array}{c} m^{\prime }\left(t\right)=f_{\text{in}}\left(t\right)-f_{\text{out}}\left(t\right) \end{array}$$ (1)

where the prime symbol stands for a derivative with respect to time *t*, *f*_*in*_ and *f*_*out*_ are respectively the sum of input (or producing) and output (or consuming) fluxes for the metabolite *m*. For cultures growing exponentially at a rate *μ*, quantities of metabolites and metabolic fluxes also increase exponentially, at the same rate *μ*. Based on this assumption, it is common practice to divide all these quantities by a biomass term *x*(*t*) = *x*(0)*e* ^*μt*^ to be able to use values that remain constant over time (hence the name “metabolic stationary state”) which we refer to using capital letters *M* = *m*/*x*, *F*_in|out_ = *f*_in|out_/*x*. After taking the derivative of *m*, we obtain

$$\begin{array}{c} \mu M=F_{\text{in}}-F_{\text{out}} \end{array}$$ (2)

The *growth* (or *dilution*, depending on the cultivation device) term *μM* is often neglected compared to *F*_in_ and/or *F*_out_. In the rest of the paper, we omit this term and consider only stationary chemostat situations. In a chemostat, the substrate is fed into the biological reactor as continuous constant flow. At the same time, the culture is pumped out of the reactor at the same flow rate, hence maintaining the volume, biomass, culture composition and a particular growth rate of the microorganism concerned, constant.

Let us consider the label balance for a metabolite with only one input and one output flux:

$$\begin{array}{c} {\left(Ml\left(t\right)\right)}^{\prime }=Fl_{\text{in}}\left(t\right)-Fl\left(t\right) \end{array}$$ (3)

where *l*(*t*) is a labeled fraction of *M* (and therefore taking its values in the interval [0, 1]), *l*_in_(*t*) is input label fraction for this metabolite and *F* is the value of the input and output fluxes, which are necessarily the same in a stationary context. As *M*′ = 0, the above equation can be simplified to:

$$\begin{array}{c} l^{\prime }\left(t\right)=\nu \left(l_{\text{in}}\left(t\right)-l\left(t\right)\right) \end{array}$$ (4)

where *ν* is a fraction *F*/*M* > 0, which is none other than a *turnover rate*. A general solution to this equation is well known and is given as

$$\begin{array}{c} l\left(t\right)=l\left(0\right)e^{-\nu t}\;+\;\nu \;\int_{0}^{t}e^{\nu \left(\tau -t\right)}l_{\text{in}}\left(\tau \right)d\tau . \end{array}$$ (5)

An analytical form of *l*(*t*) can be obtained when the integral in the Eq (5) can be taken explicitly and depends on a particular form of the input label *l*_in_(*t*). For example, when *l*_in_(*t*) is a step function, i.e. *l*_in_(*t*) = 1 for *t* ≥ 0 and *l*_in_(*t*) = 0 for *t* < 0, the solution is [14]

$$\begin{array}{c} l\left(t\right)=1-e^{-\nu t},\;\text{for }t\geq 0. \end{array}$$ (6)

Here we have assumed that *l*(0) = 0. In the following subsections, we explore a linear non-reversible pathway and labeling strategies leading to analytical forms of *l*(*t*) for all metabolites in the pathway under study. Based on these analytical forms, we will establish some basic properties of the solutions. Then, helped by the established properties, we will consider a complex network in which some fluxes will be estimated using an original numerical method in a novel context with rectangular periodic pulses.

### Label propagation in a linear pathway of non-reversible reactions under a step input signal

Let start by considering a plain linear pathway of *n* metabolites at constant concentrations *M*_*i*_ (*i* = 1, …, *n*) where all reactions are non-reversible. The constant flux across the pathway is noted *F*. At *t* = 0, the input is instantly switched from unlabeled to labeled so that the pathway receives a step input signal, i.e. *l*_in_(*t*) for the first metabolite is a step function mentioned above. All the metabolites in the pathway are assumed to be unlabeled at *t* = 0.

For the sake of simplicity, the metabolite concentrations are assumed to be all pairwise different. Later we will see that equality of different metabolite concentrations leads to singularities in solutions. Nevertheless, the cases when some concentrations are repeated (i.e. for some *i* and *j* we have *M*_*i*_ = *M*_*j*_) are correctly treated by the software accompanying this paper and the formulas for such solutions can be found in the S1 Text.

We have already given the solution for the first metabolite in Eq (6). It is known that the same function can describe the label concentration in a chemostat [15]. This can be used to interpret the first metabolite in a non-reversible pathway as a model for the label in a chemostat.

Due to the construction of the network, the label fraction of the first metabolite *l*_1_(*t*) plays the role of the source term *l*_in_(*t*) for the second metabolite and the solution of Eq (5) is

$$\begin{array}{c} l_{2}\left(t\right)=1+\frac{\nu_{2}}{\nu_{1}-\nu_{2}}e^{-\nu_{1}t}+\frac{\nu_{1}}{\nu_{2}-\nu_{1}}e^{-\nu_{2}t}. \end{array}$$ (7)

We note that this solution is a linear combination of exponential functions which makes possible to take the integral in Eq (5) in an analytical form for the next metabolite *l*_3_(*t*). A generic solution for a metabolite *i* can be written as

$$\begin{array}{c} l_{i}\left(t\right)=1+\sum_{k=1}^{i}a_{k}^{\left(i\right)}e^{-\nu_{k}t} \end{array}$$ (8)

where constant coefficients $a_{k}^{\left(i\right)}$ can be calculated simply as

$$\begin{array}{c} a_{k}^{\left(i\right)}=-\prod_{\begin{array}{l} m=1\\ m\neq k \end{array}}^{i}\frac{\nu_{m}}{\nu_{m}-\nu_{k}},k=1,\ldots ,i,\text{for }i\geq 2. \end{array}$$ (9)

or in a recursive form

$$a_{1}^{\left(1\right)}\;=-\;1$$ (10)

$$a_{k}^{\left(i\right)}=a_{k}^{\left(i-1\right)}\frac{\nu_{i}}{\nu_{i}-\nu_{k}},k=1,\ldots ,i-1,\;\text{for }\;i\;\geq \;2$$ (11)

$$a_{i}^{\left(i\right)}\;=\;-\;(1\;+\;\sum_{k=1}^{i-1}a_{k}^{\left(i\right)}).$$ (12)

Here $a_{k}^{\left(i\right)}$ designates the *k*-th exponential coefficient of the *i*-th metabolite. In our numerical experiments, we noted that due to the presence of the differences *ν*_*m*_ − *ν*_*k*_ in denominators, the absolute values of $a_{k}^{\left(i\right)}$ can increase very rapidly with an increase in the length of the pathway. This can lead to significant round off errors. Actually, only the coefficients for pathways of moderate length, say less than 10–15 metabolites, can be calculated with usual double precision arithmetic (when a floating number is represented by a storage of 64 bits). For longer pathways, arbitrary precision software is required to calculate label propagation.

Another consequence of the term *ν*_*m*_ − *ν*_*k*_ in the denominators is the need to only consider the pathways where all *M*_*i*_ (and hence all *ν*_*i*_) are pairwise different. In this way, the denominators are never zero and formula (9) can be used. As already mentioned, a more general case, when for some *m* and *k* indices it can happen that *ν*_*m*_ = *ν*_*k*_, is considered in the S1 Text.

**Fig 1:**
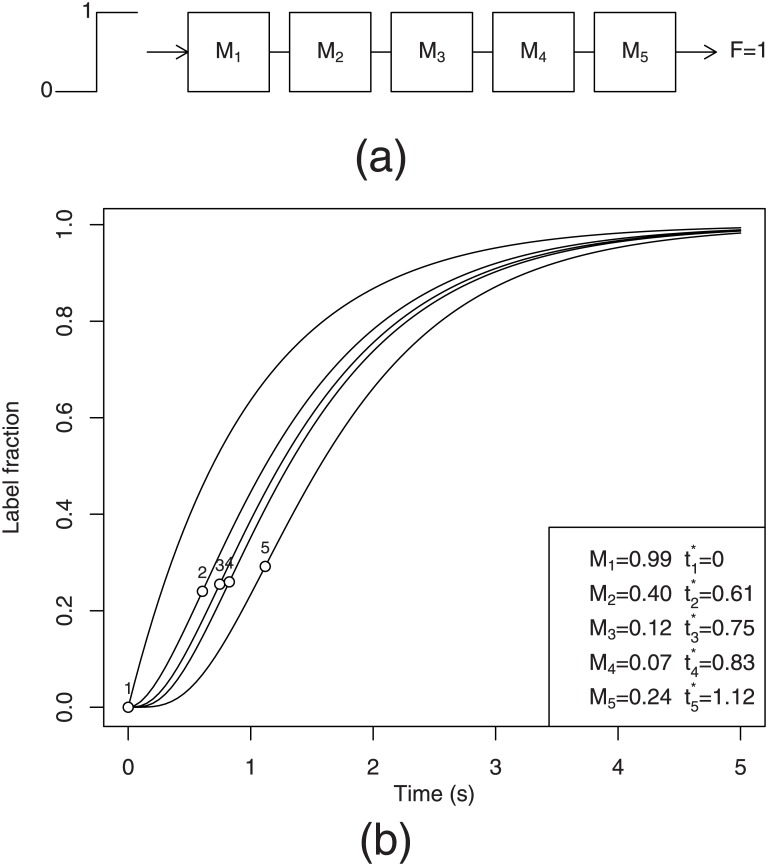
Labelling curves in a linear non-reversible pathway. (a) A linear non-reversible pathway subjected to a step label input. (b) Label propagation curves for a linear non-reversible pathway of 5 randomly sized metabolites. Circles indicate the position of “label shock wave”, i.e. the time points at which the first derivative of each labeling curve reaches maximum. The values of the pool sizes *M*_*i*_ and the shock wave positions $t_{i}^{*}$ are given in the legend.

An example of label propagation curves for a linear pathway composed of 5 metabolites is given in Fig 1. In this example, the metabolite pool sizes *M*_*i*_ were drawn randomly and uniformly from an interval [0, 1] and the flux *F* was set to 1.

We can see that the curves are sigmoid beginning at 0 (as imposed by the initial conditions) and monotonously increase to approach an asymptotic value 1. All curves except the first start with the first derivative equal to 0. As the label propagates in the network, the curves become progressively flatter. Every labeling curve evolves strictly under the curves of upstream metabolites and over the curves of the downstream metabolites. In other words, the kinetic curves can never intersect for any *t* > 0 as *l*_*j*_ (*t*) < *l*_*i*_ (*t*) for all *j* > *i*.

This statement is not specific to this particular example but results from a more general property of *coercion* which can be formally proven for any linear pathway of non-reversible reactions. To demonstrate this, let us rewrite Eq (5) after applying a rule of integration by parts and assuming *l*(0) = 0 and *l*_in_ (0) = 0:

$$\begin{array}{c} l\left(t\right)=l_{\text{in}}\left(t\right)-\;\int_{0}^{t}e^{\nu \left(\tau -t\right)}l_{\text{in}}^{\prime }\left(\tau \right)d\tau . \end{array}$$ (13)

We can see that the solution for a given metabolite *l*(*t*) is composed of its input label *l*_in_ (*t*) diminished by the value of the integral. The coercion property will be proven if it can be shown that the value of the integral is positive for any metabolite in the pathway.

Consider the solution for the second metabolite

$$l_{2}\left(t\right)=l_{1}\left(t\right)-\;\int_{0}^{t}e^{\nu \left(\tau -t\right)}l_{1}^{\prime }\left(\tau \right)d\tau$$ (14)

It can be seen from the Eq (6) that $l_{1}^{\prime }\left(t\right)>0$ for *t* > 0. So the integral in Eq (14) has a positive value and therefore *l*_2_(*t*) < *l*_1_(*t*). This inequality can be used in Eq (4) to establish that $l_{2}^{\prime }\left(t\right)>0$. In turn, adapting formula (13) to *l*_3_(*t*) implies that *l*_3_(*t*) < *l*_2_(*t*) and so on. This demonstration by induction can be repeated successively for all metabolites in the pathway. Note that it proves not only the *coercion* property but also the *monotonic increase* in labeling curves, as it was shown that $l_{i}^{\prime }\left(t\right)>0$ for all *i* = 1, …, *n*.

Another property we call *invariance under reordering* is a direct consequence of formula (9) and can be formulated as follows. The labeling curve of an *i*-th metabolite in a non-reversible linear pathway is independent of a particular order of the first *i* metabolites. Indeed, the value of a product will not change if its terms are renumbered.

The invariance under the reordering property is related to the notion of *label shock wave*(LSW) propagation. In fluid mechanics, a shock wave corresponds to abrupt changes in the characteristics of the medium. We borrow this notion to describe abrupt changes in label fraction to draw a parallel between the propagation of the label in a pathway and the propagation of a shock wave. Naturally, LSW positions (noted $t_{i}^{*}$ for *i* = 1, …, *n*) can be represented by the points of maximum first derivatives (indicated by circles in Fig 1) as they correspond to the points where the maximum speed of label change has been reached. The value $t_{1}^{*}$ is always 0 as the maximum derivative of the first metabolite is reached at *t* = 0. This can be easily checked by taking the first derivative of Eq (6). An analytical expression for $t_{2}^{*}$ can be obtained by taking the second derivative of Eq (7) and finding the time point $t_{2}^{*}$ at which the second derivative equals 0:

$$\begin{array}{c} t_{2}^{*}=\frac{\ln\nu_{2}-\ln\nu_{1}}{\nu_{2}-\nu_{1}} \end{array}$$ (15)

It is not easy to obtain a general analytical expression for the LSW position of an *i*-th metabolite. For $t_{3}^{*}$, the following empirical approximation was established

$$\begin{array}{c} t_{3}^{*}≃t_{2}^{*}+\frac{\ln\nu_{3}-\ln\nu_{1}}{\nu_{3}-\nu_{1}} \end{array}$$ (16)

For the following metabolites, the delay between two successive LSW positions *i* − 1 and *i* (for *i* ≥ 4) is approximated by νi−1=MiF. The longer the *residence time* νi−1=MiF, the longer the LSW will be delayed by the *i*-th metabolite:

$$\begin{array}{c} t_{i}^{*}≃t_{3}^{*}+\sum_{k=4}^{i}\nu_{k}^{-1}\;\text{for}\;i=4,\ldots ,n \end{array}$$ (17)

**Fig 2:**
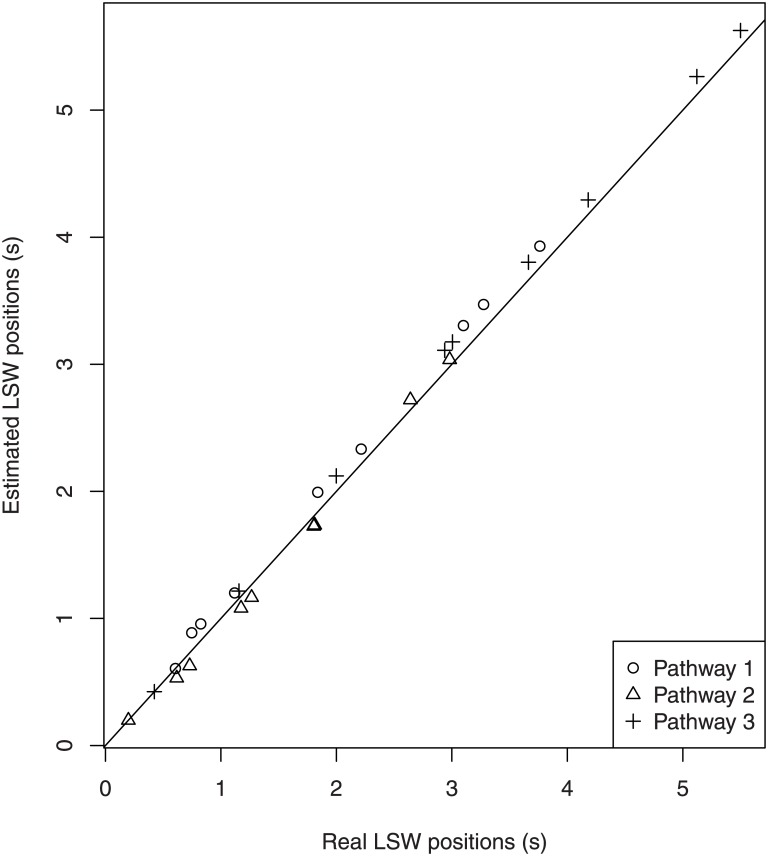
Comparison between estimated and real LSW positions. We now compare three randomly generated linear non-reversible pathways of length 10. Estimations were based on formula (17). Real values were obtained by numerically solving $l_{i}^{\prime \prime }\left(t\right)=0$. The solid line corresponds to *y* = *x*.

An agreement between estimated and exact LSW positions is illustrated in Fig 2. We can see that estimations of $t_{i}^{*}$ based on formulas (15–17) are close enough to the real LSW positions found numerically for three randomly generated linear non-reversible pathways of length 10. The fact that in some pathways the LSW positions lie on a line parallel to the line *y* = *x* means that LSW are well approximated for all metabolites except one or two. These one or two over- or underestimated LSW positions introduce a shift in positions for all the following metabolites in the pathway.

Invariance of the sum with respect of metabolite ordering in formula (17) is in agreement with the invariance under reordering established above. However, the latter property is a stronger affirmation as it postulates that not only the LSW of the last metabolite in the pathway will remain in the same position under metabolite shuffling but also that the whole labeling curve of the last metabolite will be exactly the same irrespective of the order of metabolites. On the other hand, LSW position for the last metabolite is relatively independent of particular values of *M*_*i*_/*F* under the condition that their sum is kept the same.

A practical consequence of the invariance property is that if we observe only the label of the last metabolite in a pathway, there is no unique way to estimate all {*ν*_*i*_}_*i* = 1, …, *n*_. Another practical consequence is related to metabolite lumping in a linear non-reversible pathway. If, for the sake of network simplicity, several metabolites are lumped together to form a new fictitious metabolite, the pool size of this new metabolite should equal the sum of lumped pools. In this way, the LSW position will be kept more or less at the same moment in time.

The time needed for an LSW to pass through the entire pathway can be used as a measure of the pathway length from the point of view of labeling. The longer the whole LSW delay, the longer the pathway for labeling.

A parameter, called the relaxation time of a pathway *T*_*r*_, can be introduced to characterize the duration of the label propagation in the network. The parameter is defined as the time needed for all metabolites in the pathway to be labeled at a level greater or equal to 0.99 of the input label fraction. This time is always finite. It can be seen by examining the structure of the solution in Eq (8). Indeed, we can always find a finite time when all exponents terms are low enough to sum up to an absolute value less then 0.01. This parameter will be useful in the following subsections.

### Sinusoidal label input

So far, we have considered a linear non-reversible pathway subjected to a step label input. Let us consider now the same pathway subjected to a sinusoidal label input. The characterization of a system by application of sinusoidal input signals and analysis of its outputs is a common practice in many disciplines, for example in electronics or more generally in studies of automatic systems, including some biological molecular systems [16]. To date, such an approach has not been applied to isotope labeling experiments. However, experimental devices allowing the sinusoidal addition of compounds are described in the literature, e.g., [17] meaning the application of sinusoidal label inputs is experimentally feasible.

Let us start by recalling that sine and cosine functions can be represented as a linear combination of exponential functions with imaginary exponents:

$$\begin{array}{c} \cos\left(\omega t\right)=\frac{e^{i\omega t}+e^{-i\omega t}}{2},\;\;\;\;\sin\left(\omega t\right)=\frac{e^{i\omega t}-e^{-i\omega t}}{2i}. \end{array}$$ (18)

Here, *i* is the imaginary unit and *ω* is an angular frequency. Another way to deal with a sinusoidal wave is to keep only a real or imaginary part of *e* ^*iωt*^. Without loss of generality, let us consider only cos(*ωt*) in the input signal. To keep it between 0 and 1, a constant must be added. To obtain the maximum possible wave amplitude, a wave amplitude of 0.5 and an additive constant of the same value were chosen, so that the input signal looks like

$$\begin{array}{c} l_{in}\left(t\right)=\frac{1+\cos(\omega t+\pi )}{2} \end{array}$$ (19)

A phase delay *π* was added to make the input signal continuous at *t* = 0. This was done only to obtain more aesthetic graphs and is not essential for the subsequent developments. Indeed, a constant phase delay *ψ* does not change the way to represent a cosine as exponential, it just makes the amplitude a complex number:

$$\begin{array}{c} a\cos\left(\omega t+\psi \right)=\text{Re}\left(ae^{i(\omega t+\psi )}\right)=\text{Re}\left(ae^{i\psi }e^{i\omega t}\right) \end{array}$$ (20)

so that the new complex amplitude of the exponential is the constant product *ae* ^*iψ*^.

In previous subsections, a generic solution for label inputs written as a linear combination of exponential with real coefficients was established. Now, the above considerations show the interest of generalizing the input label to a linear combination of exponential functions with complex exponents and coefficients:

$$\begin{array}{c} l_{in}\left(t\right)=\sum_{k=1}^{n_{0}}a_{k}^{\left(0\right)}e^{\beta_{k}t},\;\;\text{for }t\geq 0 \end{array}$$ (21)

here the exponents *β*_*k*_ are complex numbers, as are the coefficients $a_{k}^{\left(0\right)}$, while *n*_0_ is an integer giving the number of different exponential terms in the input signal. For example, the step label input considered in the previous subsection fits this formula by setting *n*_0_ = 1, $a_{1}^{\left(0\right)}=1$ and *β*_1_ = 0. It is worth noting that formula (21) can be used to approximate any real world periodic signal whatever its waveform. More will be said about such representations in the next subsection.

The linear nature of Eq (5) offers us the opportunity to consider every input exponential separately and then to sum the results in a final solution with appropriate coefficients. Let us examine the contribution to the solution of an isolated complex sinusoidal wave *l*_*in*_(*t*) = *e* ^*iωt*^. By taking the integral in Eq (13) we get the output label

$$\begin{array}{c} l\left(t\right)=\frac{\nu }{\nu +i\omega }\left(e^{i\omega t}-e^{-\nu t}\right) \end{array}$$ (22)

We can see that the solution is composed of a sinusoidal wave *e* ^*iωt*^ and a decaying exponential *e* ^−*νt*^. After a relaxation period (as defined in the previous subsection), the term *e* ^−*νt*^ will become negligeable and only the stationary wave part of the *l*(*t*) will be observable. In practice, such a stationary wave solution could facilitate the collection of labeled samples. Another possible advantage is obtaining technical replicates to reduce measurement errors. Finally, in resolving the inverse problem of fitting parameters, it is much easier to fit a stationary sinusoidal wave than a linear combination of exponential functions that we saw in the case of step input signal. We will discuss the possible advantages of the periodic input label in more detail at the end of the last section of this paper.

Let us now look more closely at the wave part of solution Eq (22). After passing through a metabolite pool, the wave kept its frequency *ω* but changed its amplitude and phase. The amplitude is damped by a factor |νν+iω|=vv2+ω2<1 while the phase is shifted by *ψ* = Arg(*ν/ν*+*iω*) = arctan(−*ω/ν*) ∈ (−*π*/2, 0) which corresponds to a delay *d* = *ψ/ω* ≃ −1/*ν* in wave propagation. Note that the delay is basically the same as that found empirically in the step label experiment. The amplitude is always diminished whatever the values of *ν* and *ω* while the phase shift is always negative, i.e. the wave is always damped and delayed, and never “advanced”. These two properties ensure that the solution Eq (22) is physically realistic.

**Fig 3:**
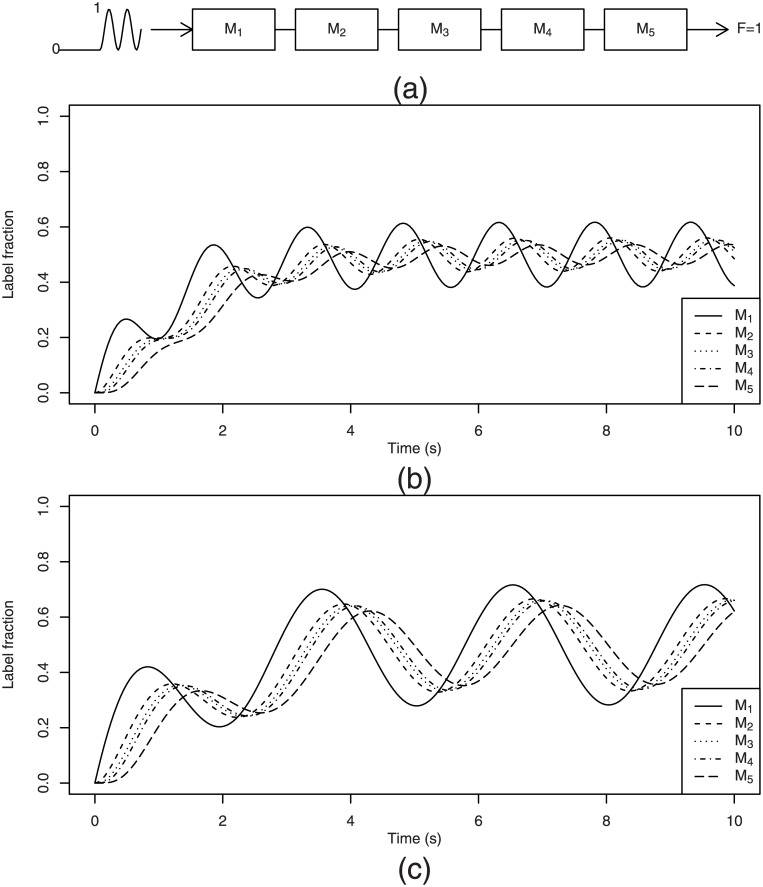
Examples of label propagation in the pathway of Fig 1 after applying a sinusoidal Eq (19) label input. (a) Pathway scheme subjected to a sinusoidal label input; (b) A high frequency input wave (*T* = 2*π/ω* = 1 s) leads to a rapid reduction in amplitude through propagation via the pathway. (c) A low frequency (*T* = 2 s) preserves the wave amplitude at the end of the pathway better and leaves the wave delay almost unchanged compared to case *T* = 1 s. The same wave delay explains the impression that this graph is merely stretched out compared to graph b) (abstraction made of the change in amplitude).

It is interesting to note that the reduction in the amplitude is greater for bigger *ω* but the delay is less sensitive to *ω* variations, especially when *ω/ν* ≪ 1. These observations are important for the choice of an appropriate input frequency. An example of simulations with high and low input frequencies are presented in Fig 3.

A generic solution for the label of *i*-th metabolite that resulted from input Eq (21) is given by

$$\begin{array}{c} l_{i}\left(t\right)=\sum_{k=1}^{n_{0}}a_{k}^{\left(0,i\right)}e^{\beta_{k}t}+\sum_{k=1}^{i}a_{k}^{\left(i\right)}e^{-\nu_{k}t} \end{array}$$ (23)

where the constant coefficients $a_{k}^{\left(0,i\right)}$ and $a_{k}^{\left(i\right)}$ can be calculated according to

$$a_{k}^{\left(0,i\right)}=a_{k}^{\left(0\right)}\prod_{m=1}^{i}\frac{\nu_{m}}{\nu_{m}+\beta_{k}},\;k\;=\;1,\;\ldots ,\;n_{0}$$ (24)

$$a_{1}^{\left(1\right)}\;=\;-\sum_{k=1}^{n_{0}}a_{k}^{\left(0,1\right)},$$ (25)

$$a_{k}^{\left(i\right)}\;=\;a_{1}^{\left(1\right)}\prod_{\begin{array}{c} m=1\\ m\neq k \end{array}}^{i}\frac{\nu_{m}}{\nu_{m}-\nu_{k}},\;i>1.$$ (26)

These formulas generalize formula (9) and the previous observations made concerning a single stationary wave: the decaying exponentials *e* ^−*ν*_*k*_*t*^ will vanish after a certain relaxation time *T*_*r*_ and the label will have only a stationary wave part corresponding to a superposition of exponentials that have purely imaginary exponents *β*_*k*_. If some *β*_*k*_ are not purely imaginary, i.e. they have a negative real part, they will be naturally damped because *e* ^*Re*(*β*_*k*_)*t*^ → 0 as *t* → ∞. Note that positive real parts in *β*_*k*_ are not physically realizable as this would have led to values tending to infinity as *t* → ∞.

The structure of formulas (24–26) shows that the invariance under reordering established for the step input also holds for a more general input Eq (21) as the products in Eqs (24 and 26) are independent of the order of the metabolites in the pathway up to the rank *i*. Hence, the shape of the labeling curve of the *i*-th metabolite in a linear non-reversible pathway will remain unaffected if the lower or equal ranked metabolites are reordered.

### Rectangular periodic pulses

In the previous subsection, we saw that a sinusoidal input could have some practical advantages for dynamic labeling experiments but is difficult to implement from a technical point of view. In this subsection, we show that applying RPP inputs can avoid such technical difficulties without jeopardizing the advantages of sinusoidal inputs.

RPP are relatively simple to perform in a chemostat culture. At *t* = 0, an input is switched from unlabeled to a labeled substrate and maintained for an interval of time *t*_1_. At *t* = *t*_1_, the input is switched back to the unlabeled substrate and maintained for an interval of time *t*_2_. At *t* = *t*_1_ + *t*_2_, the substrate is again switched to the labeled input, and so on. A full labeling cycle lasts *T* = *t*_1_ + *t*_2_.

From a theoretical point of view, the solution for the first labeled phase of RPP is the same as the one described in subsection for the step input. But the formulas given in the subsection should be modified for the following RPP periods, as the metabolites are already labeled at the beginning of each period, i.e. the previous assumption of unlabeled state as initial condition does not hold for the pulses which follow the first one.

To obtain the formulas for intervals *t*_1_ or *t*_2_, we place the time reference *t* = 0 at the beginning of each interval and use the solution at the end of the previous interval as the initial label state for the interval concerned. The whole solution will simply be the juxtaposition of piece-wise solutions in labeled/unlabeled intervals.

The solution for labeled and unlabeled intervals is still a linear combination of exponential functions like in Eq (8). But in this case, the initial label states *L*_*i*_ = *l*_*i*_(0) ≠ 0 is taken into account. Modified recursive formulas for $a_{k}^{\left(i\right)}$ during a labeled period *t*_1_ are

$$a_{1}^{\left(1\right)}=L_{1}-1$$ (27)

$$a_{k}^{\left(i\right)}=a_{k}^{\left(i-1\right)}\frac{\nu_{i}}{\nu_{i}-\nu_{k}},\;k=2,\;\ldots ,\;i-1$$ (28)

$$a_{i}^{\left(i\right)}=L_{i}-(1+\sum_{k=1}^{i-1}a_{k}^{\left(i\right)})$$ (29)

During an unlabeled interval, the solutions can be written as

$$\begin{array}{c} l_{i}\left(t\right)=\sum_{k=1}^{i}a_{k}^{\left(i\right)}e^{-\nu_{k}t}, \end{array}$$ (30)

which tends to 0 as *t* → ∞ and not to 1 as was the case in Eq (8). For the unlabeled interval, the coefficients can be calculated according to

$$a_{1}^{\left(1\right)}=L_{1}$$ (31)

$$a_{k}^{\left(i\right)}=a_{k}^{\left(i-1\right)}\frac{\nu_{i}}{\nu_{i}-\nu_{k}},k=1,\;\ldots ,\;i-1,\text{for }i\geq 2$$ (32)

$$a_{i}^{\left(i\right)}=L_{i}-\sum_{k=1}^{i-1}a_{k}^{\left(i\right)}$$ (33)

**Fig 4:**
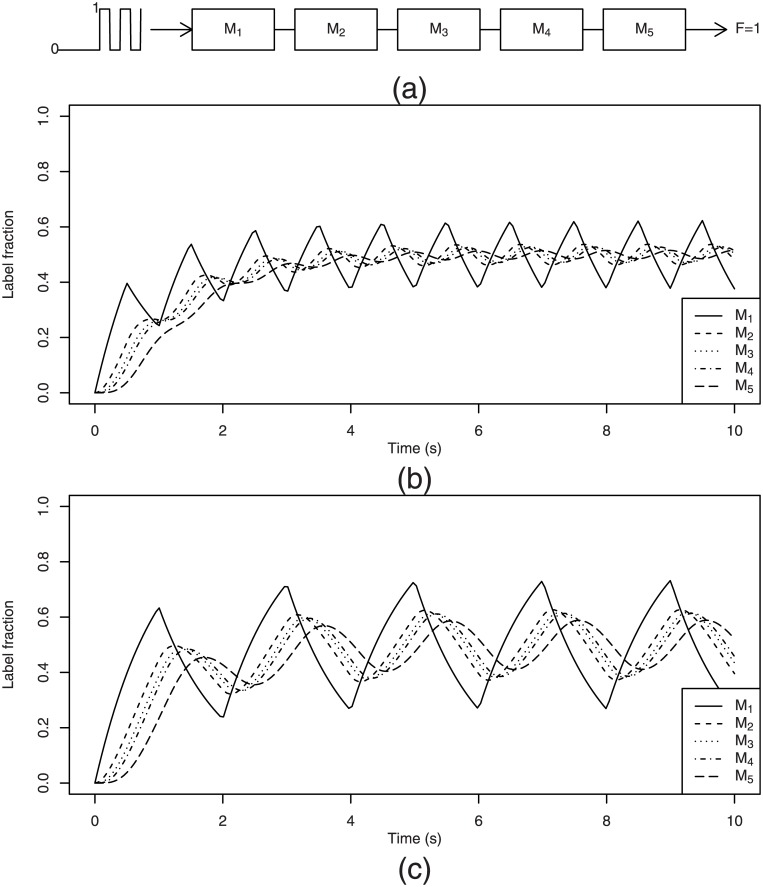
Examples of label propagation in the previously considered linear pathway subjected to an RPP label input. The labeling curves of metabolites ranked 3 or above closely resemble the labeling curves obtained with a plain sinusoidal wave input. (a) Pathway subjected to an RPP label input; (b) Like in sinusoidal wave input, applying an RPP with high frequency (*T* = 1 s) leads to a rapid reduction in amplitude as the signal moves along the pathway. (c) The same observation as for sinusoidal wave applies to the low frequency RPP (*T* = 2 s): it preserves the wave amplitude at the end of the pathway better and leaves the wave delay observed for *T* = 1 s almost unchanged.

Two examples of numerical simulations with two different periods *T* = 2*π/ω* of 1 and 2 seconds are presented in Fig 4. The periods *T* in these examples were chosen to be the same as in the two examples given in Fig 3 for sinusoidal inputs. We can see that after application of an RPP input, the labeling curves of the metabolites furthest from the label entry point (in this example, metabolites with rank 3 or above) look very similar to those obtained for a sinusoidal wave input. This is a logical consequence of the fact that high frequencies are damped faster than low frequencies.

Indeed, each real world periodic function, such as RPP, can be approximated by an appropriate linear combination of sine and cosine functions with periods that are multiples of *T* by using the Fourier series. Each individual wave in this series will behave according to principles established in the previous subsection: after going through each metabolite pool, the wave amplitude will be reduced to a greater or lesser extent and its phase delayed by different length periods depending on its frequency and on the turnover rate *ν* of the crossed metabolite. After a relaxation time *T*_*r*_, independent of frequencies, a stationary periodic solution will be set up for each metabolite. Its particular shape will depend on how each particular Fourier component (called *harmonic*) was damped and delayed. The further we move towards the end of the pathway, the more the harmonic with the lowest frequency will dominate. It will make the signal look increasingly like a plain sinusoidal wave with a period *T*.

In this subsection, we have proposed a solution obtained by the juxtaposition of step labeling. But there is an alternative way to obtain the stationary wave part of such a solution. Fourier series corresponding to periodic input signals can be found and the formulas (23–26) can be applied to each harmonic in this series, which can then be summed to obtain the resulting stationary wave form. Let us stress that this approach can be applied to any periodic label input, e.g., ramp waveforms or alike. In the following subsection, a simplified version of this strategy is applied to a complex metabolic network.

### Inverse problem in RPP labeling experiments

Up to now, our discussion focused on the direct problem: knowing the values of fluxes and of the metabolite pools, simulate label propagation in a linear non-reversible pathway or more generally, in a metabolic network. In this subsection, we are also interested in the inverse problem: given a network and knowing some (noisy) label propagation curves, how to estimate fluxes and metabolite pools whose label kinetics match these curves. Usually, inverse problems are solved in a least squares framework where free parameters (here a *ν* vector, a ratio of fluxes and pool sizes) are adjusted iteratively to create simulations to fit experimental measurements.

It follows from formulas (24–26) that parameters of an observable periodic signal are particularly easy to evaluate if we know {*ν*_*i*_}_*i* = 1, …, *n*_. Actually, only the parameters of the first harmonic are simulated, as the other harmonics will be more or less damped after crossing a few metabolites. Even if the final signal does not yet look like a plain sinusoidal wave, we fit a single sinusoidal wave of period *T* and use its parameters as experimental data to be fitted. This major simplification of experimental data appears to be sufficient for the practical purpose of estimating fluxes in the example below.

It turns out that the straightforward formulas (24–26) used to estimate harmonic parameters in a linear pathway also hold for a general case of any metabolic network. To confirm this, let us consider a linear system of equations describing label propagation in terms of cumomers (cumulated isotopomers) of weight 1 [18, 19]. Given that cumomers of weight 1 and EMU of weight 1 are equivalent entities, for the sake of brevity, we use only the term “cumomers”. In this framework, the ODE linear system is

$$\begin{array}{c} l^{\prime }\left(t\right)=Al\left(t\right)+s\left(t\right) \end{array}$$ (34)

with a *n* × *n* real matrix *A* (which only depends on the turnover rate vector *ν*) expressing the cumomer balance, and complex vectors *l*(*t*) and *s*(*t*) of the length *n*, which are respectively cumomer and input (or source) vectors. Taking into account only source terms of a given frequency *ω*: *s*(*t*) = *be* ^*iωt*^ and looking for a solution in stationary wave form *l*(*t*) = *ae* ^*iωt*^ (where *a* and *b* are constant complex vectors of length *n*), we get a new linear system on unknowns *a* after simplifying by *e* ^*iωt*^

$$\begin{array}{c} i\omega a=Aa+b, \end{array}$$ (35)

which is solved as

$$\begin{array}{c} a=-{\left(A-i\omega I\right)}^{-1}b. \end{array}$$ (36)

Here *I* is a *n* × *n* identity matrix. Under certain conditions (no isolated sub-network, no zero flux through a metabolite), all eigenvalues of the matrix *A* are known to have a strictly negative real part [20], which means that the matrix *A* − *iωI* is invertible under the same conditions. The fact that a single matrix inversion is sufficient to simulate the vector *a* from known *A* and *b* (instead of a full ODE system solution) is of major importance, since it enables a radical simplification of parameter estimation in the inverse problem in the RPP labeling experiment.

**Fig 5:**
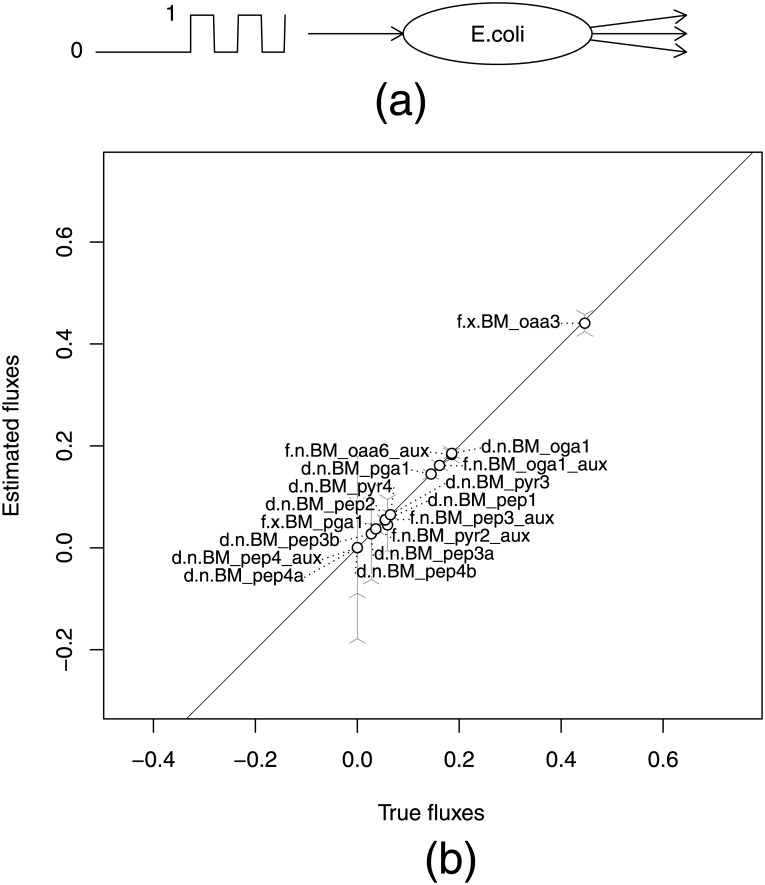
Comparison of estimated and true fluxes in a simulated RPP experiment. (a) Scheme of RPP experiment on *E. coli*. Fully labeled glucose is fed to the culture in RPP form. Several aminoacids are measured by GC-MS. (b) Only statistically and structurally identifiable free and dependent fluxes (distinguished by “f” and “d” at the beginning of the flux names) are reported (17 of 70). The prefixes “n” and “x” in flux names correspond to net and exchange fluxes respectively. Exchange fluxes are mapped on [0, 1] intervals. Error bars correspond to 95% CI estimated by linearized statistics. The solid line indicates *y* = *x* positions. This example shows that in an RPP experiment, we can expect a good agreement between true and estimated values for identifiable fluxes.

We illustrate the feasibility of this approach by simulating the propagation of a label in *Escherichia coli* metabolism upon addition of uniformly labeled glucose according to RPP label input. The metabolic network was taken from Supplementary Data 3 of [21] corresponding to the reference network from the Fig 5 of the cited paper. It is defined in an FTBL file which stands for Flux Table format. This format was introduced in [3] and originally used as input format for 13CFlux software. In this plain text file, several sections describe the network topology, measurement data and some additional information. We used only the parts describing the network topology, the nature of fluxes (reversible or not) and what kind of molecules and their fragments were measurable. As to measured labeling data, they were modeled by a numerical ODE solution of system Eq (34). The labeling data considered in these simulations were expressed as ‘mean label incorporation’ *m*(*t*) representing the fraction of label in the molecular species. For a *k*-th metabolite *M*_*k*_, this value can be calculated in two different but equivalent ways.

The first way is a simple averaging of cumomers of weight 1

$$\begin{array}{c} m_{k}=\frac{\sum_{i=1}^{n_{k}^{\left(c\right)}}l_{ik}}{n_{k}^{\left(c\right)}}, \end{array}$$ (37)

where *l*_*ik*_ is a *i*-th cumomers of weight 1 of the *k*-th metabolite, $n_{k}^{\left(c\right)}$ is the number of carbon atoms in the *k*-th metabolite and *m*_*k*_ its mean label. This formula is used for simulations in the direct problem.

Another way to calculate the mean label is to pass by a weighted sum of mass isotopomers *M*_*k*,+*i*_ ($i=0,\ldots ,n_{k}^{\left(c\right)}$), e.g., *M*_*k*,+0_ is a fraction of molecules of metabolite *M*_*k*_ with 0 labeled carbon atoms, *M*_*k*,+1_ is a fraction of molecules with only one labeled carbon atom, and so on [22, 23]

$$\begin{array}{c} m_{k}=\frac{\sum_{i=1}^{n_{k}^{\left(c\right)}}iM_{k,+i}}{n_{k}^{\left(c\right)}} \end{array}$$ (38)

The second way is used to estimate mean label values from the mass spectrometry (MS) measurements. A formal proof of the equivalence of formulas (37) and (38) is given in the S2 Text.

## Results and Discussion

In this section, we present a numerical example estimating fluxes of central carbon metabolism of *E. coli*. The numerical method underlying this example is based on the subsection “Inverse problem in RPP labeling experiments”. For the sake of brevity, we don’t detail it here but invite an interested reader to examine the accompanying software distributed under OpenSource licence. We have voluntary limited numerical examples to this alone case leaving linear non reversible chains aside because of its practical interest and its closeness to real world problems.

The simulations of experimental data were done with arbitrarily chosen but realistic fluxes and metabolic pool sizes. Metabolite fragments observable in MS were the same as in the original FTBL file [21], i.e. 24 amino acid fragments resulting from 11 distinct amino acids. Note that using uniformly labeled substrate as sole carbon source makes the data redundant as the mean label on any fragment of a molecule is the same as the mean label for the whole molecule. We kept all the fragments in the original dataset to stay close to real experimental conditions and because redundant measurements make it possible to reduce the confidence intervals (CI) of estimated parameters. All metabolic pool sizes were considered as known. After an ODE simulation of the label propagation in a RPP experiment with *t*_1_ = *t*_2_ = 30 min and “sampling” at 2-min intervals, a centered Gaussian noise with a standard deviation of 0.01 was added to the mean label to simulate an experimental noise. The chemostat was simulated as running for a period of 10 hours and the data were “sampled” during the last hour.

A least squares problem was formulated as minimizing the difference between the Fourier coefficients for the first harmonic of measured metabolites and the harmonic fitted to the simulated experimental noisy data. In this problem, 28 free variables (2 Fourier parameters for the input harmonic plus 26 free fluxes) were estimated by fitting 2 × 24 = 48 experimental data (one harmonic for each of 24 metabolite fragments and 2 coefficients per harmonic) of which only 2 × 11 = 22 are not redundant. This problem is therefore structurally undetermined because the number of independent measurements (22) is less than the number of parameters to estimate (28). Its Jacobian matrix (i.e. the matrix of partial derivatives of the residual vector with respect to the parameter vector) is necessarily rank deficient. The least squares problem was solved using the NLSIC algorithm with an additional requirement of least norm increments [6]. The starting point for the optimization was randomly chosen.

In Fig 5, the estimated flux values obtained by solving the least squares problem are presented and compared to their true values which were used in the ODE simulation. Note that only 17 out of a total 70 free and dependent fluxes that were structurally and statistically identifiable are shown. These 17 fluxes (7 free fluxes and 10 dependent fluxes) were selected on the basis of 95% CI below ±0.2. The full set of fitted fluxes can be found in S3 Text.

This example is by no means an approximation of a real experiment. Too many shortcuts were used in the network topology. For example in the cited FTBL file, the synthesis of serine is resumed just as PGA → Ser (3-phospho-D-glycerate → L-serine) while, in reality, this transformation includes two more intermediate compounds: 3-phospho-hydroxypyruvate and 3-phospho-L-serine. Such shortcuts are commonly accepted for stationary labeling as they have absolutely no impact on measurements but they do introduce more or less perceptible biases for the dynamic label propagation. This example simply shows that an RPP approach can be successfully applied to realistic complex networks including linear pathways, reversible reactions, branching, condensing reactions and cycles.

Among the many numerical advantages offered by RPP inputs, we can emphasize the very low computational requirements both for memory and CPU time; independence of the calculus procedure on an actual form of input periodic signal; noise robustness and last but not least, ease of Jacobian calculation.

Some drawbacks are common to all dynamic labeling methods. It is frequently true that in a poorly defined network or because of missing data, not all fluxes can be estimated. Fortunately, most of those that can are in good agreement with true values. Another drawback is the tight coupling between fluxes *f* and pool sizes *M* meaning that label data can be used only to estimate turnover rate vector *ν*, but not to estimate *f* and *M* distinctly. This is why the label data must be accompanied by some metabolite and/or flux measurements to assess all the fluxes and metabolites. In the example above, we assumed that *M* was known, which allowed us to estimate the remaining part of *ν*, the flux vector *f*.

The potential technical advantages of RPP inputs include the following features:

**autocheck** for a stationary periodic regime. As two consecutive labeling cycles must provide almost identical signals, it is easy to see whether a desired stationary regime is achieved or not. If only one cycle is sampled, two halves of the cycle must be symmetrical in a stationary regime (provided that *t*_1_ = *t*_2_);
**outliers** in measurements are identified more easily in a periodic waveform;
**sample accumulation** is allowed during consecutive label cycles. In a chemostat, the use of large collection volumes can significantly affect the stability of the regime. Hence, collecting a series of small samples rather than a single large sample is much better to ensure the physiological state of the cells remains constant. Moreover, measurement errors can be reduced by increasing the size of the sample and/or accumulating the quantity of samples;
**the resolution of sampling frequency** can be enhanced if the sampling time grids are made with bigger time steps but are shifted to interleave from one labeling cycle to another. Traditionally, the sampling of labeled material in dynamic labeling experiments is very challenging, because samples have to be collected very rapidly (within seconds) in sufficient amounts, and at high sampling frequency (depending on the metabolic timescales). By spreading the sample collection over several cycles and interleaving them, RPP label input facilitates sampling from a practical point of view.

## Conclusions

The theoretical investigations reported here introduce novel concepts in the description of label propagation, such as label shock wave and its delay, relaxation time and stationary wave. Depending on the shape of the label input, some basic properties of the label propagation were established. Curve coercion, monotonic increase and invariance under reordering (or in a weaker form: LSW delay invariance) were established for step inputs, while wave damping and phase delay were explicitly formulated for periodic inputs. All these developments will help advance our understanding and rational interpretation of labeling data in real experiments, but also help develop a more general numerical approach for the simulation of dynamic labeling data in the near future. These new concepts also enable better identification of the strengths, pitfalls and limitations of the dynamic labeling, thereby enabling better prediction of the situations in which it could be applied successfully.

This paper also introduced periodic inputs of label as highly valuable alternatives to classical step inputs. Compared to other input shapes (step label and sinusoidal wave), rectangular periodic pulses are very attractive not only because they give reliable estimations of fluxes for a realistic network with very low computational effort, but also because they are relatively simple to perform experimentally and offer valuable practical advantages in labeled metabolite sampling.

## Supporting Information

S1 Text Repeated metabolite pools in a linear non-reversible pathway. Formulas for label dynamics in a linear non-reversible pathway are generalized to a case when some metabolites have exactly the same pool sizes.
(PDF)

S2 Text Equivalence of two ways for calculating mean label. A formal proof of this equivalence is provided.
(PDF)

S3 Text Fitting results of *E. coli* network in RPP labeling. Full set of fitted fluxes with reference and precision data.
(PDF)

S1 Software Simulating label propagation. R script with necessary data and parameters to reproduce all figures of the paper.
(ZIP)
