# Supplementary material for: Theoretical Basis for Dynamic Label Propagation in Stationary Metabolic Networks under Step and Periodic Inputs
Source: PLoS One. 2015 Dec 7;10(12):e0144652. doi: 10.1371/journal.pone.0144652 (PMC4671543; doi:10.1371/journal.pone.0144652)
Supplement: S1 Text — Formulas for label dynamics in a linear non-reversible pathway are generalized to a case when some metabolites have exactly the same pool sizes. (PDF) [file pone.0144652.s001.pdf]

Theoretical basis for dynamic label propagation in stationary metabolic networks under step and periodic inputs

Supplementary Information

S1 File

Repeated metabolite pools in a linear non-reversible pathway

Let us start with a simple case of a linear non-reversible pathway composed of two metabolic pools of identical size $M_1 = M_2 = M$ subjected to a step label input at $t = 0$ with all the metabolites in unlabeled state at this moment in

time. The label kinetics of the first metabolite is already known (6)[1]

$$l_1(t) = 1 - e^{-\nu t}.$$

To find $l_2(t)$, we take an integral in equation (5)

$$l_2(t) = \nu \int\limits_0^t e^{\nu(\tau - t)} l_1(\tau) d\tau = \nu e^{-\nu t} \int\limits_0^t e^{\nu\tau}(1 - e^{-\nu\tau}) d\tau = \nu e^{-\nu t} \int\limits_0^t (e^{\nu\tau} - 1) d\tau =$$

$$= e^{-\nu t}(e^{\nu\tau} - \nu\tau)\Big|_0^t = e^{-\nu t}(e^{\nu t} - \nu t - 1) = 1 - (1 + \nu t)e^{-\nu t}\ .$$

We can see a polynomial factor $1 + \nu t$ appears next to the exponential $e^{-\nu t}$. In the case of a longer pathway composed of n identical metabolite pool sizes, the solution for n-th metabolite label includes a polynomial of degree $n - 1$ multiplied by the same exponential

$$l_n(t) = 1 - e^{-\nu t} \sum_{i=0}^{n-1} \frac{(\nu t)^i}{i!}$$

This makes it necessary to review the generic solution (8) to take into account the possible appearance of polynomials instead of simple constant factors for different exponentials. Let us now introduce the following notations:

$\{\lambda_j\}_{j=1,\ldots,n_i}$ a vector of *distinct* values of ν_k for $k = 1, \ldots, i$, where n_i indicates how many distinct values of ν_k constitute the vector λ;

$\delta_{ij} = \nu_i - \lambda_j$;

[1] In this document, equation numbers refer to the original paper.

$P(t,\, a) = \sum_{i=0}^{n} \frac{a_i t^i}{i!}$ is a polynomial of variable t with a real or complex vector of coefficients $\{a_i\}_{i=0,\ldots,n}$ which are normalized by the corresponding factorials $i!$ (n is the degree of the polynomial);

$a_j^{(i)}$ is a vector of normalized polynomial coefficients of a metabolite i for the term $e^{-\lambda_j t}$. We note $N_j^{(i)}$ the degree of $P(t,\, a_j^{(i)})$. In the case of the step label input, all polynomial coefficients are real numbers. Obviously, the vector $a_j^{(i)}$ defines the polynomial $P(t,\, a_j^{(i)})$ in a unique way.

With these notations, the generic solution of the i-th label in a linear non-reversible pathway, unlabeled at $t = 0$ and subjected to the step label input, can be written in the following form

$$l_i(t) = 1 - \sum_{j=1}^{n_i} P(t,\, a_j^{(i)}) e^{-\lambda_j t}$$

We provide recursive formulas for calculating $a_j^{(i)}$ vectors from already known ones for the metabolites ranking from 1 to $i-1$. The recursion starts with a polynomial for $i = 1$ with a vector of coefficients composed of only the term $a_1^{(1)} = \{1\}$.

The recursive formulas are given for two cases: 1) when the newly considered metabolite has ν_i which is different from λ_j so that $\delta_{ij} \neq 0$ and 2) when one exists among the preceding metabolites (let its number be j) such that $\lambda_j = \nu_i$.

For the first case ($\lambda_j \neq \nu_i$), the formula for a k-th component of the vector $a_j^{(i)}$ is

$$a_{jk}^{(i)} = a_{jk}^{(i-1)} + \sum_{l=k}^{N_j^{(i-1)}} (-\lambda_j) a_{jl}^{(i-1)} (-\delta_{ij})^{k-l-1} + \sum_{l=k}^{N_j^{(i-1)}-1} a_{jl+1}^{(i-1)} (-\delta_{ij})^{k-l-1}$$

for $k = 0, ..., N_j^{(i-1)}$. Note that the second sum must be ignored when $k = N_j^{(i-1)}$. As the value ν_i is not present among λ_j for all $j = 1, ..., N_j^{(i-1)}$ then there is a newly created term for $\lambda_{j+1} = \nu_i$, and for which we have

$$a_{j+1\,0}^{(i)} = - \sum_{l=1}^{N_j^{(i-1)}} a_{l0}^{(i)}.$$

This new polynomial is introduced with a degree 0 so that $n_i = n_{i-1} + 1$, $N_{n_i}^{(i)} = 0$ and $N_j^{(i)} = N_j^{(i-1)}$ for all $j = 1, ..., n_{i-1}$.

For the second case ($\lambda_j = \nu_i$):

$$\begin{aligned} a_{j0}^{(i)} &= 1 \\ a_{jk}^{(i)} &= \lambda_j a_{jk-1}^{(i-1)} \text{ for } k = 1, ..., N_j^{(i-1)} + 1. \end{aligned}$$

These formulas are implemented in the software accompanying the paper. Even more, the formulas implemented in the software are generalized for the case when metabolites in the pathway are authorized to be already labeled at time $t = 0$. The corresponding algorithms can be found in the file `labprop.inc.R` in the function `linpoly_exp()`.
