# Supplementary material for: Theoretical Basis for Dynamic Label Propagation in Stationary Metabolic Networks under Step and Periodic Inputs
Source: PLoS One. 2015 Dec 7;10(12):e0144652. doi: 10.1371/journal.pone.0144652 (PMC4671543; doi:10.1371/journal.pone.0144652)
Supplement: S2 Text — A formal proof of this equivalence is provided. (PDF) [file pone.0144652.s002.pdf]

Theoretical basis for isotopic label propagation in stationary metabolic networks under step and periodic inputs

Supplementary Information

S2 File

Equivalence of two ways for calculating mean label

In this appendix, a formal proof of the equivalence of formulas (37)[1] and (38) is given.

Let us consider a metabolite with n carbon atoms. Its 2^n isotopomer

[1] In this document, equation numbers refer to the original paper.

fractions are noted $\{x_{0...0},\ x_{0...1,},...,\ x_{1...1}\}$ where n-positional binary indexes are used with a usual convention that 1 corresponds to labeled carbon and 0 to unlabeled carbon. The binary label mask of length n can be interpreted as binary writing of an integer number not exceeding 2^n. This fact is used in decimal indexing of isotopomers $\{x_i\}_{i=0,...,2^n-1}$. According to the definition of label fractions, we can write

$$\sum_{i=0}^{2^n-1} x_i \equiv 1$$

A binary n-positional mask of a decimal integer i is noted $b_n(i)$, e.g. $b_5(2) = 00010$. If the context is not ambiguous, the index n can be omitted.

Cumomers of weight 1 for the metabolite concerned (also known as positional label fractions) are defined in agreement with [1]

$$l_p = \sum_{b(i)\&b(2^{p-1})\neq 0} x_i \text{ for } p = 1, ..., n$$

where indexes i run through all values from 0 to $2^n - 1$ for which a binary operator & (called *and*-operator) in the expression $b(i)\&b(2^{p-1})$ gives a non zero value. In other words, all x_i for which the binary mask of i has a 1 in the p-th position are summed.

Mass isotopomers are noted M_{+i} and formally defined as

$$M_{+i} = \sum_{s_1(b(j))=i} x_j \text{ for any } i = 0, ..., n$$

here $s_1(b(i))$ means a sum of 1's in the binary mask $b(i)$. For the sake of brevity, we write $s_1(i)$. So, M_{+i} includes all x_j whose binary mask has exactly i 1's or equivalently, exactly i labeled carbon atoms.

In this notation, the property which has to be proved is

$$\sum_{p=1}^{n} l_p = \sum_{i=1}^{n} iM_{+i}$$

The left part sum is noted S.

Let us take some decimal index k in $0, ..., 2^n - 1$ and see how many times the label fraction x_k is accounted for in S. By definition, x_k is included in a l_p iff $b(i)\&b(2^{p-1}) \neq 0$, in other words, iff the binary mask $b(k)$ includes a 1 in the p-th position. There are exactly $s_1(k)$ such l_p terms in S. Therefore, x_k is present $s_1(k)$ times in S.

Let us now run the index k through the indexes included in the M_{+i} for a given i. For each k from this set, we can see that x_k is present i times in S as $s_1(k) \equiv i$. Summing all the x_k concerned, we see that M_{+i} is present i times in S.

Repeating this procedure for all $i = 1, ..., n$ and noting that all terms x_k in S were accounted for in these procedures, the equivalence property is proved.

References

[1] Wiechert W, Möllney M, Isermann N, Wurzel M, de Graaf AA. Bidirectional reaction steps in metabolic networks: III. Explicit solution and analysis of isotopomer labeling systems. Biotechnol Bioeng. 1999;66(2):69–85.
