# Supplementary material for: Theoretical Basis for Dynamic Label Propagation in Stationary Metabolic Networks under Step and Periodic Inputs
Source: PLoS One. 2015 Dec 7;10(12):e0144652. doi: 10.1371/journal.pone.0144652 (PMC4671543; doi:10.1371/journal.pone.0144652)
Supplement: S3 Text — Full set of fitted fluxes with reference and precision data. (PDF) [file pone.0144652.s003.pdf]

Theoretical basis for dynamic label propagation in stationary metabolic networks under step and periodic inputs

Supplementary Information

S3 File

Fitting results of *E. coli* network in RPP labeling

In this file, a complete list of fluxes is presented, both fitted and constrained. They can be compared with true flux values that were used to produce simulated data. The flux names are formed according to the following pattern [dfc].[nx].NAME. The letters “d”, “f” and “c” mean “dependent”, “free” and

"constrained" respectively. The letters "n" and "x" stand for "net" and "exchange" fluxes respectively. The exchange fluxes have been mapped on [0,1) interval. The confidence interval (CI) reported in the table corresponds to 95% confidence. Fluxes are labeled as identifiable if their CI<=0.2. As constrained fluxes are not fitted, their CI is assigned to ±NA (non available) and the notion of indetifiablity is not applicable to them, hence NA in the column "Identifiable".

The data presented in the table below are also available in electronic form in R (www.r-project.org) environment. To get access to them, install the software accompanying the paper, launch R in the installation directory and execute:

```
> load('figures.RData')
```

after loading saved data, the table is available in a dataframe called `df`.

Names	True	Estimated	Estimated-True	95% CI	Identifiable
d.n.BM_oaa1	1e-04	0.002	0.001925	±2.21	no
d.n.BM_oaa3	1e-04	-0.5418	-0.541867	±2189.44	no
d.n.BM_oaa3_aux	0	-0.5418	-0.541767	±2190.86	no
d.n.BM_oaa4	1e-04	0	-1e-04	±2.23	no
d.n.BM_oaa4_aux	1e-04	0	-1e-04	±2.23	no
d.n.BM_oaa6a	0.0924	0.0915	-0.000962	±1.11	no
d.n.BM_oaa6b	0.0924	0.0915	-0.000962	±1.11	no
d.n.BM_oga1	0.1852	0.1852	0	±0	yes

Names	True	Estimated	Estimated-True	95% CI	Identifiable
d.n.BM_oga2	0.024	0.0235	-0.000547	±0.46	no
d.n.BM_oga2_aux	0.024	0.0235	-0.000547	±0.46	no
d.n.BM_pep1	0.0548	0.0548	0	±0	yes
d.n.BM_pep2	0.0548	0.0548	0	±0	yes
d.n.BM_pep3a	0.0273	0.0273	-1.5e-05	±0.09	yes
d.n.BM_pep3b	0.0273	0.0273	-1.5e-05	±0.09	yes
d.n.BM_pep4_aux	2e-04	2e-04	3e-05	±0.18	yes
d.n.BM_pep4a	1e-04	1e-04	1.5e-05	±0.09	yes
d.n.BM_pep4b	1e-04	1e-04	1.5e-05	±0.09	yes
d.n.BM_pga1	0.1446	0.1446	0	±0	yes
d.n.BM_pyr2	0.0361	0.0371	0.001012	±0.51	no
d.n.BM_pyr3	0.065	0.065	0	±0	yes
d.n.BM_pyr4	0.065	0.065	0	±0	yes
d.n.ana2	-3.4481	-6.5955	-3.147406	±20859634472.67	no
d.n.ana3	5.9336	6.0997	0.166198	±10378.12	no
d.n.coOut	8.8766	8.8756	-0.001012	±0.51	no
d.n.emp2	2.1622	1.6169	-0.545249	±455411603.26	no
d.n.emp3	2.1621	1.6169	-0.545149	±9412.29	no
d.n.emp4	4.3131	3.7679	-0.545149	±9412.29	no
d.n.emp5	4.1196	3.5745	-0.545149	±9412.29	no
d.n.gs1	2.3385	-0.087	-2.425536	±432.09	no

Names	True	Estimated	Estimated-True	95% CI	Identifiable
d.n.gs2	2.3385	-0.087	-2.425536	±432.09	no
d.n.ppp1	0.1814	1.8168	1.635446	±28236.88	no
d.n.ppp2	0.0586	1.1489	1.090297	±18824.59	no
d.n.ppp3	0.1228	0.6679	0.545149	±9412.29	no
d.n.ppp4	0.0019	0.547	0.545149	±9412.29	no
d.n.ppp5	0.0567	0.6019	0.545149	±9412.29	no
d.n.ppp6	0.0567	0.6019	0.545149	±9412.29	no
d.n.tca2	2.8666	2.3215	-0.545149	±9412.29	no
d.n.tca3	0.5281	2.4085	1.880387	±9840.04	no
d.n.tca4	0.3347	2.2151	1.880387	±9840.04	no
d.n.tca5a	1.3366	1.0641	-0.272574	±4706.15	no
d.n.tca5b	1.3366	1.0641	-0.272574	±4706.15	no
d.n.tca6	-0.9218	-4.0587	-3.136882	±15364.03	no
d.n.upt0	0	0	0	±0	yes
f.n.BM_oaa1_aux	0	0.5438	0.543792	±7.29	no
f.n.BM_oaa6_aux	0.1848	0.1829	-0.001925	±0.01	yes
f.n.BM_oga1_aux	0.1612	0.1618	0.000547	±0	yes
f.n.BM_pep3_aux	0.0546	0.0546	-3e-05	±0	yes
f.n.BM_pyr2_aux	0.0361	0.0371	0.001012	±0	yes
f.n.ana1	0.5557	0	-0.555673	±69437378.83	no
f.n.emp1	2.1121	0.4767	-1.635446	±93.99	no

Names	True	Estimated	Estimated-True	95% CI	Identifiable
f.n.emp6	5	3.8233	-1.17671	±48.89	no
f.n.gneo1	1e-04	0	-1e-04	±1515957.89	no
f.n.gneo2	5	6.9602	1.960172	±43.02	no
f.n.tca1	5.4967	2.526	-2.970684	±29.91	no
c.n.BM_Ac	0.2266	0.2266	0	±NA	NA
c.n.BM_F6P	0.0086	0.0086	0	±NA	NA
c.n.BM_G6P	0.0065	0.0065	0	±NA	NA
c.n.BM_GAP	0.0129	0.0129	0	±NA	NA
c.n.BM_OAA	0.0304	0.0304	0	±NA	NA
c.n.BM_OGA	0.0082	0.0082	0	±NA	NA
c.n.BM_PEP	0.0062	0.0062	0	±NA	NA
c.n.BM_PGA	0.0488	0.0488	0	±NA	NA
c.n.BM_PYR	0.0497	0.0497	0	±NA	NA
c.n.BM_R5P	0.0661	0.0661	0	±NA	NA
c.n.BM_pga1_aux	0.1446	0.1446	0	±NA	NA
c.n.BM_pyr4_aux	0.065	0.065	0	±NA	NA
c.n.upt	2.3	2.3	0	±NA	NA
c.n.uptU	2.3	2.3	0	±NA	NA
d.x.tca5a	0.945	0.1131	-0.831882	±4044.65	no
f.x.BM_oaa1	0.6965	0.744	0.047475	±0.62	no
f.x.BM_oaa3	0.4464	0.4406	-0.005768	±0.02	yes

Names	True	Estimated	Estimated-True	95% CI	Identifiable
f.x.BM_oga1	0.9779	0.3156	-0.662279	±0.86	no
f.x.BM_pga1	0.0592	0.0448	-0.014406	±0.05	yes
f.x.emp1	0.1	0.99	0.89	±196147.56	no
f.x.emp3	0	0	0	±26388782.54	no
f.x.emp4	0.6072	0	-0.607158	±29880.14	no
f.x.emp5	0.9	0.1977	-0.702334	±34.81	no
f.x.ppp2	0	0	0	±1022911.77	no
f.x.ppp3	0	0	0	±69404335.03	no
f.x.ppp4	0.5381	0.99	0.451896	±305720.33	no
f.x.ppp5	0	0	0	±110414.03	no
f.x.ppp6	0.8434	0	-0.843358	±317860.86	no
f.x.tca5b	0.945	0.1131	-0.831882	±13.46	no
f.x.tca6	0.1	0	-0.1	±152.06	no
c.x.BM_Ac	0	0	0	±NA	NA
c.x.BM_F6P	0	0	0	±NA	NA
c.x.BM_G6P	0	0	0	±NA	NA
c.x.BM_GAP	0	0	0	±NA	NA
c.x.BM_OAA	0	0	0	±NA	NA
c.x.BM_OGA	0	0	0	±NA	NA
c.x.BM_PEP	0	0	0	±NA	NA
c.x.BM_PGA	0	0	0	±NA	NA

Names	True	Estimated	Estimated-True	95% CI	Identifiable
c.x.BM_PYR	0	0	0	±NA	NA
c.x.BM_R5P	0	0	0	±NA	NA
c.x.BM_oaa1_aux	0	0	0	±NA	NA
c.x.BM_oaa3_aux	0	0	0	±NA	NA
c.x.BM_oaa4	0	0	0	±NA	NA
c.x.BM_oaa4_aux	0	0	0	±NA	NA
c.x.BM_oaa6_aux	0	0	0	±NA	NA
c.x.BM_oaa6a	0	0	0	±NA	NA
c.x.BM_oaa6b	0	0	0	±NA	NA
c.x.BM_oga1_aux	0	0	0	±NA	NA
c.x.BM_oga2	0	0	0	±NA	NA
c.x.BM_oga2_aux	0	0	0	±NA	NA
c.x.BM_pep1	0	0	0	±NA	NA
c.x.BM_pep2	0	0	0	±NA	NA
c.x.BM_pep3_aux	0	0	0	±NA	NA
c.x.BM_pep3a	0	0	0	±NA	NA
c.x.BM_pep3b	0	0	0	±NA	NA
c.x.BM_pep4_aux	0	0	0	±NA	NA
c.x.BM_pep4a	0	0	0	±NA	NA
c.x.BM_pep4b	0	0	0	±NA	NA
c.x.BM_pga1_aux	0	0	0	±NA	NA

Names	True	Estimated	Estimated-True	95% CI	Identifiable
c.x.BM_pyr2	0	0	0	±NA	NA
c.x.BM_pyr2_aux	0	0	0	±NA	NA
c.x.BM_pyr3	0	0	0	±NA	NA
c.x.BM_pyr4	0	0	0	±NA	NA
c.x.BM_pyr4_aux	0	0	0	±NA	NA
c.x.ana1	0	0	0	±NA	NA
c.x.ana2	0	0	0	±NA	NA
c.x.ana3	0	0	0	±NA	NA
c.x.coOut	0	0	0	±NA	NA
c.x.emp2	0	0	0	±NA	NA
c.x.emp6	0	0	0	±NA	NA
c.x.gneo1	0	0	0	±NA	NA
c.x.gneo2	0	0	0	±NA	NA
c.x.gs1	0	0	0	±NA	NA
c.x.gs2	0	0	0	±NA	NA
c.x.ppp1	0	0	0	±NA	NA
c.x.tca1	0	0	0	±NA	NA
c.x.tca2	0	0	0	±NA	NA
c.x.tca3	0	0	0	±NA	NA
c.x.tca4	0	0	0	±NA	NA
c.x.upt	0	0	0	±NA	NA

Names	True	Estimated	Estimated-True	95% CI	Identifiable
c.x.upt0	0	0	0	±NA	NA
c.x.uptU	0	0	0	±NA	NA
